# Intrinsic Osteoinductivity of Porous Titanium Scaffold for Bone Tissue Engineering

**DOI:** 10.1155/2017/5093063

**Published:** 2017-07-26

**Authors:** Maryam Tamaddon, Sorousheh Samizadeh, Ling Wang, Gordon Blunn, Chaozong Liu

**Affiliations:** ^1^Institute of Orthopaedic & Musculoskeletal Science, University College London, Royal National Orthopaedic Hospital, Stanmore HA7 4LP, UK; ^2^State Key Laboratory for Manufacturing System Engineering, Xi'an Jiaotong University, Xi'an, Shanxi Province 710049, China

## Abstract

Large bone defects and nonunions are serious complications that are caused by extensive trauma or tumour. As traditional therapies fail to repair these critical-sized defects, tissue engineering scaffolds can be used to regenerate the damaged tissue. Highly porous titanium scaffolds, produced by selective laser sintering with mechanical properties in range of trabecular bone (compressive strength 35 MPa and modulus 73 MPa), can be used in these orthopaedic applications, if a stable mechanical fixation is provided. Hydroxyapatite coatings are generally considered essential and/or beneficial for bone formation; however, debonding of the coatings is one of the main concerns. We hypothesised that the titanium scaffolds have an intrinsic potential to induce bone formation without the need for a hydroxyapatite coating. In this paper, titanium scaffolds coated with hydroxyapatite using electrochemical method were fabricated and osteoinductivity of coated and noncoated scaffolds was compared in vitro. Alizarin Red quantification confirmed osteogenesis independent of coating. Bone formation and ingrowth into the titanium scaffolds were evaluated in sheep stifle joints. The examinations after 3 months revealed 70% bone ingrowth into the scaffold confirming its osteoinductive capacity. It is shown that the developed titanium scaffold has an intrinsic capacity for bone formation and is a suitable scaffold for bone tissue engineering.

## 1. Introduction

Massive traumatic injuries or tumour resections are among the factors which can contribute to substantial bone loss [1, 2]. Thanks to a spontaneous capacity for regeneration, most bone lesions, such as fractures, can be repaired with conventional therapies. The process of fracture healing is a sequence that begins with hematoma formation and then moves to inflammation, destruction of nonvital debris, granulation tissue proliferation, callus formation, conversion of woven bone to lamellar bone, and, finally, remodelling of the healed bone [3]. However, in cases of large defects and osseous congenital deformities, bone grafts (e.g., xeno-, allo-, and autografts) or substitutes are needed to aid healing [4]. The current gold standard for repair of large bone defects [1] is autograft where host bone is removed from another non-load-bearing site to fill the defect. However, the complication rate is as high as 30% due to donor site morbidity, pain, hematoma, and inflammation. In many cases, this has been proven a challenging treatment for critical-sized defects [1].

Tissue engineering (TE) approaches, which use body's natural ability to repair injured bone with new bone tissue and to remodel newly produced bone in response to the local stresses, are being explored as alternatives for large bone defect repairs [5]. There are three key ingredients necessary in TE: a scaffold, which may be either natural or synthetic, cells [6], and inductive signals (i.e., growth factors or proteins) [7].

Studies have suggested that cells might be unable to establish themselves properly within a defect without matrix guidance [8]. Therefore, a scaffold must be developed to provide a three-dimensional structure to support the cells, aid their proliferation, and help them be differentiated, while its architecture defines the ultimate shape of the new bone [9, 10].

In addition to general requirements for TE scaffolds such as biocompatibility and ability to be sterilised, the key requirements for the development of an orthopaedic scaffold include the following [1]:

1. Mechanical stability to be retained in the affected area
2. Interconnected porous architecture (porosity exceeding 90%) [4, 11] to allow for vascularization and bone ingrowth and to act as a channel for delivery of nutrients and gases to the cells deep inside the scaffold and, at the same time, removal of the metabolic waste from cells
3. Supporting and promoting osteogenic differentiation of undifferentiated cells (osteoinduction) and growth of differentiated bone cells (osteoconduction) [12]
4. Enhancing cellular activity towards scaffold-host tissue integration (osseointegration).

Mechanical properties are especially important in scaffolds for hard and ductile tissues such as bone [13, 14] because the scaffolds must also interact with their physiological surroundings to transmit mechanical signals to cells and regulate cell behaviour (i.e., differentiation, motility, and contractility). The stiffness of scaffold can have effects at a transcriptional level, determining whether stem cells make the decision to become cells as functionally diverse as osteoblasts [15].

Biomaterials used in tissue engineering of bone are usually categorized into four major groups: natural polymers, synthetic polymers, metallic materials, and inorganic materials such as ceramics and bioactive glasses. Multicomponent systems can be designed to generate composites of enhanced performance [16].

Naturally derived polymers have the advantage of native biological function [17, 18] but their low mechanical strength makes them less attractive as an option for bone tissue repair. In synthetic polymers, on the other hand, it is possible to precisely control the mechanical properties; however, they exhibit poor cell adhesion [18]. Bioceramics are known to enhance and promote biomineralization [14, 16], but their brittleness and low fracture toughness means that they are mostly suitable only in combination with other materials and in form of composites.

Metallic scaffolds are promising alternatives for hard load-bearing tissue repairs. These biomaterials in their solid form have been widely used for fabrication of the implants replacing hard human tissues for many years [19], and therefore in their porous form they can be possible candidates for TE approaches. Titanium and its alloys are of great interest in biomedical applications due to their excellent combinations of mechanical properties, biocompatibility, and chemical stability [20] and one of their drawbacks, which is the mismatch of mechanical properties between bulk titanium and natural bone which leads to stress shielding, and eventual implant loosening, that can be rectified by producing a low modulus porous network [21, 22]. In fact, Ti meshes have been successfully used in spine fusion surgeries and for oral and maxillofacial structures [23]. Introduction of porosity and pore interconnectivity improves mechanical fixation and osteointegration by allowing extensive body fluid transport through the porous implant. This can provoke bone tissue ingrowth, consequently leading to the development of a stable interface between the scaffold and host tissue [19].

The porous Ti matrices can be produced using techniques such as powder metallurgy (PM) [19, 22] or additive manufacturing technique (AM) [20, 24]. The drawbacks of the PM including limited control over the size, shape, and distribution of the porosity [25] can be resolved using AM techniques. Selective laser melting (SLM) and Selective Laser Sintering (SLS) are two of the AM processes that are able to produce complex structures layer by layer with high precision. Where SLS uses a very precise nanolaser beam to sinter the powder material to build up the structure [24], SLM fully melts the powder to form a solid mass. Direct metal laser sintering (DMLS) is essentially similar to SLS in terms of method, as it involves sintering rather than melting, but where SLS is used for polymers, ceramics as well as metals, DMLS is used exclusively for metals. With DMLS it is possible to control the porosity of each layer, pore interconnectivity, size, shape, and distribution, and consequently the 3D architecture of the implant, by changing the processing parameters, such as laser power, laser spot diameter, and layer thickness, or by modifying the size of the original titanium particles [26, 27].

Titanium is considered a bioinert material, which does not possess osteoconductivity or osteoinductivity by itself; however, the surface can be modified to induce osteoconductivity [28] or osteoinductivity [29]; hydroxyapatite (HAp) is a very good biomaterial which has excellent osteoinductivity and has been widely used in bone defect repairs. HAp coatings can be used to prompt osteogenesis without the need for additional osteogenic cells or bone morphogenic proteins (BMP) [28]. Conventional HAp coating of solid titanium surfaces involves plasma spraying which is a line of sight technique and fails to coat inner surfaces of porous structures. HAp coatings can be produced using techniques such as electrochemical deposition and biomimetic method. Both methods are based on precipitation from aqueous solutions, take place at low temperature, are economical, and allow coating of complex shapes. The biomimetic method uses simulated body fluids (SBF) that mimic physiological ionic strength and pH. In a typical electrochemical deposition, a precursor (brushite) is first formed that is converted into hydroxyapatite (HA) through an ageing process. Thus this method offers a control over crystallinity [30]. However, debonding and loss of HAp coatings in vivo are one of the main concerns in using these coatings [31, 32].

In this study, we have produced a porous titanium scaffold for bone tissue engineering using SLS technique. We have used both electrochemical and biomimetic processes to coat the three-dimensional Ti matrix with hydroxyapatite and compared the osteoinductivity of coated and noncoated scaffolds in vitro. Electrochemical deposition leads to a more uniform coating and was used as the main coating method for any further analysis. Our results showed that interestingly HAp coating did not significantly increase osteogenecity (Alizarin Red production) in vitro and noncoated Ti scaffolds were also osteoinductive. These scaffolds were then implanted in sheep femoral condyle to investigate bone formation and ingrowth. Extensive osteoinduction and osteointegration (70% bone ingrowth) were observed in vivo, confirming the intrinsic capacity of the produced porous Ti scaffolds for bone regeneration.

## 2. Materials and Methods

### 2.1. Fabrication of Ti Matrix

Titanium lattices were fabricated from commercially pure titanium powder (cp-Ti) using a DMLS system (EO SINTM270). Cp-Ti and titanium alloys (typically Ti6Al4V) are used extensively as dental and orthopaedic implants, respectively. Alloying improves the mechanical properties of titanium for use in high load-bearing applications; however, some concerns related to the toxicity of various alloying elements do exist [33]. Since no significant differences were observed in terms of osseointegration, biomechanical anchorage, and bacterial interaction between cp-Ti and Ti alloys [33], we selected cp-Ti (grade 4, 99% purity, density 4.51 g/cm^3^, and Young's modulus of 105 GPa) in this study to avoid this concern.

A 200 W Yb fiber laser was used to sinter Ti powder. The scaffold was built at a speed of 4 mm^2^/s with layer thickness of 40 *μ*m, resulting in a cylindrical scaffold (10 mm × 8 mm) with strut thickness of 1.5 mm, pitch size of 0.75 mm, and porosity of 72%.

Prior to coating, the scaffolds were ultrasonically cleaned in 10% Decon®90 (Decon Laboratory Limited, UK), distilled water, and ethanol for 15 mins each, and dried in air.

### 2.2. HAp Coating Deposition and Characterisation

Two methods for HAp coating of porous Ti matrix were employed: biomimetic coating process and electrochemical deposition. In the biomimetic coating procedure, saturated simulated body fluid (5x SBF) was used according to a previously published method [30]. Briefly, coating solutions A and B were prepared (Table 1) by dissolving reagent grade salts (Sigma-Aldrich, UK) at 37°C with a constant 5% CO_2_ supply and stirring in distilled water. Samples were firstly soaked in solution A, for 24 or 48 hrs at 37°C and then in solution B for 48 hrs at 40°C with constant stirring.

In the electrochemical method, titanium lattices were immersed in the CaP solution (0.13 M, Ca(H_2_PO_4_)_2_, Sigma-Aldrich, UK) and attached to the negative terminal of a DC Dual Power Supply pack (Peak Tech, Telonic Instruments Ltd, UK). Two different electrical current densities of 10 and 6 mA/cm^2^ were applied between the two electrodes for 10 mins. The samples were then soaked in 0.1 M NaOH solution for 72 h, cleaned in distilled water, and air-dried. Scaffold formulations are summarized in Table 2.

Morphology of deposited HAp was observed by scanning electron microscopy (SEM, JEOL JSM 5500 LV, at 10 kV) and elemental analysis was performed by energy dispersive X-ray spectroscopy (EDAX, EDAX Inc., USA).

### 2.3. Evaluation of Mechanical Properties and In Vitro Mechanical Stability

The mechanical properties of the structures were determined in a universal material testing machine (Instron® 5565) under uniaxial compressive load. Ti scaffolds (*n* = 3) were placed between two hard metal compression inserts. The force and deformation were recorded during the strain-controlled compression phase with a constant strain of 1 mm/min at room temperature.

**Figure 1:**
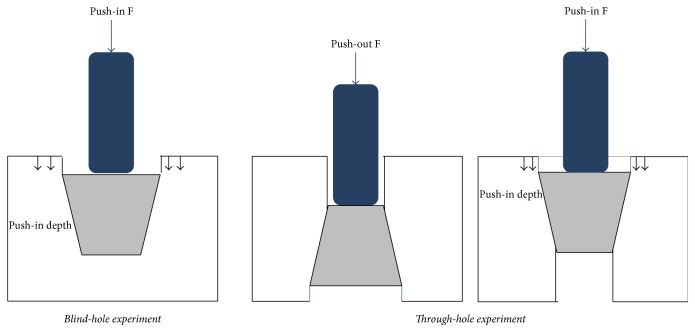
Schematics of blind and through holes experiments.

Mechanical stability of scaffolds in a defect site was examined using mechanical push-in and push-out tests (*n* = 3) in dry state in Sawbones©. Tapered cylindrical defects matching the dimensions of the scaffolds were made using appropriate drill bits in Sawbones© polyurethane foam (Sawbones Europe AB, Malmö, Sweden). We used Sawbones© foam with a density of 160 kg/m^3^ and a compressive modulus of 66 MPa comparable to that of cancellous bone [34]. For push-in/push-out tests, two types of experiments were designed: blind-hole experiment to determine the push-in depth and strength, and a through-hole experiment to observe the push-out and interfacial strengths. Samples were inserted into the created defects in Sawbones©, and the load required to push them in/out (a ramp compressive extension of 1 mm/min) was monitored. The schematic illustration of each setup is depicted in Figure 1. Based on the maximum load achieved (from the load-displacement curves) and the area of scaffolds in contact with Sawbones© (lateral surface area of a truncated cone × 28% scaffold density) the interface strength between the scaffolds and their surroundings was calculated.

### 2.4. In Vitro Evaluation of Osteoinductivity

Viability and osteoinductivity of sheep bone marrow mesenchymal stem cells (BMMSCs) were evaluated on HAP coated and noncoated Ti scaffolds. BMMSCs were isolated from sheep bone marrow aspirate, expanded, and maintained in tissue culture flasks containing Dulbecco's modified eagles medium (DMEM, Sigma-Aldrich, UK) supplemented with 10% fetal calf serum (FCS, First Link, UK) and 100 Units/mL of Penicillin and Streptomycin (P/S, Gibco, UK). Flasks were kept at 37°C with 5% CO_2_ and passaged when 80% of confluency was reached. BMMSCs were characterised by demonstrating their multipotency by differentiating them down the adipogenic, chondrogenic, and osteogenic lineages.

Three sample groups were tested: coated Ti scaffold (EM10), noncoated Ti scaffold, and Thermanox™ discs (Nalge Nunc International, USA) as control (*n* = 3) in osteogenic media.

Samples were sterilised by autoclaving and were then seeded with 10,000 BMMSCs (passage 3) in a total volume of 50 *μ*L basal cell culture media (DMEM, 10% FCS, 1% P/S). After incubation for 1 hr at 37°C with 5% CO_2_, 2-3 mL of osteogenic (basal media with 0.1 lM Dexamethasone, 500 lM Ascorbic Acid, and 10 mM b-glycerophosphate; all from Sigma-Aldrich, UK) cell culture media was added to each well. Media were changed every 3–5 days. Cell adhesion and morphology (by DAPI and phalloidin stainings at days 7 and 21), proliferation (by AlamarBlue© activity at days 1, 14, and 28), and differentiation into the osteogenic lineage (Alizarin Red staining at day 28) and cell colonisation and morphology (SEM at day 21) were studied for all groups and controls.

To assess cell adhesion and morphology, scaffolds were fixed in 4% paraformaldehyde (PFA) for 30 min, washed twice with PBS, and permeabilised with 0.25% (v/v) Triton X-100 in PBS for 30 min. Samples were blocked with 3% (w/v) bovine serum albumin (BSA) in PBS for 30 min and the actin cytoskeleton was stained with Alexa Fluor 568 phalloidin (Invitrogen; 1 : 200) for 1 h. Nuclei were stained with DAPI (4,6-Diamidino-2-phenylindole, dihydrochloride, Sigma; Fluoroshield) for 1 min. Samples were mounted on glass slides and cells were observed under a ZEISS ApoTome.2 Fluorescent Microscope (ZEISS, Germany).

AlamarBlue© was used to examine the proliferation of cells on the samples. AlamarBlue© (AbD Serotec, UK) was diluted in phenol free DMEM (Sigma, UK) to make a 10% working solution. Samples were washed with PBS and incubated with 1 mL of the working solution at 37°C and 5% CO_2_. After 4 h, 200 *μ*L from each sample was loaded in triplicate into a FluoroNunc™ white 96-well plate and fluorescence was measured at 530–560 nm excitation and 590 nm emission using a microplate reader (Infinite® 200 PRO, Tecan, Switzerland). Results were compared to those of an empty well loaded with 1 mL of the working solution at the beginning of the assay.

To assess late stage of osteogenesis, Alizarin Red staining was performed quantitatively. At day 28, samples were fixed with 4% (w/v) PFA for 30 mins and then washed with PBS. Samples were then incubated at room temperature with Alizarin Red solution for 30 mins, after which they were washed with PBS and incubated with 200 *µ*l 10% CPC in 10 mM sodium phosphate (pH 7) for 15 mins. Duplicates of 100 *µ*l of supernatant were transferred to a Nunc® 96-well plate, and the absorbance was measured with a microplate reader (Infinite 200 PRO, Tecan, Switzerland) at 570 nm.

### 2.5. In Vivo Evaluations of Mechanical and Biological Fixation

The bone tissue reaction to the porous Ti scaffold was examined by animal tests using sheep condyle. Three sheep (77–82 kg) were sedated by intravenous administration of ketamine and midazolam and sedation was maintained using gaseous anaesthesia with 2.5% isoflurane. The sheep were given Ceporex injections (active ingredient cephalexin), an antibiotic, on days 0, 1, 2, and 3. Each sheep also had fentanyl patches on days 1 and 3.

A collagen type-I–HAp scaffold was fabricated to act as the control using a freeze-drying method. Briefly, lyophilised collagen powder (Sigma-Aldrich, UK) was dispersed in distilled water (pH 3.2 with acetic acid) using IKA blender while HAp powder (Sigma-Aldrich, UK) was added to the mix. The suspension was then casted into 3D printed resin moulds, frozen, and freeze-dried. The noncoated Ti scaffolds, as well as collagen-HAp scaffolds, were implanted into 10 mm (depth and upper diameter) bone defects in the left medial condyle of sheep stifle joints and fixed by press-fit only. The limbs were scanned radiologically and implanted titanium scaffold and surrounding tissue were retrieved 12 weeks after operation. Micro-computed tomography (micro-CT) analysis was performed on samples using a Nikon XT H 225 with 110 kVP X-ray source and 112 mA (resolution of ~18–22 *μ*m) in order to assess the new bone formed within the titanium matrix. Three-dimensional reconstructions were performed using Nikon CT Agent. Subsequent visualization and analysis were performed in Bruker Software CTVOX and CTAN. Subchondral bone repair was expressed as percentage bone volume over the total volume (% BV/TV), while SEM (JEOL JSM 5500 LV, at 10 kV) observations were used to assess bone-scaffold interface.

### 2.6. Statistical Analysis

Statistical analyses were performed using OriginPro 2015 (OriginLab). The data are presented as means with standard deviation. One-way ANOVA and subsequent post hoc Tukey tests were used to analyze differences among the groups at significant level of 0.05. 

## 3. Results and Discussion

### 3.1. Characterisation of HAp Coating

**Figure 2:**
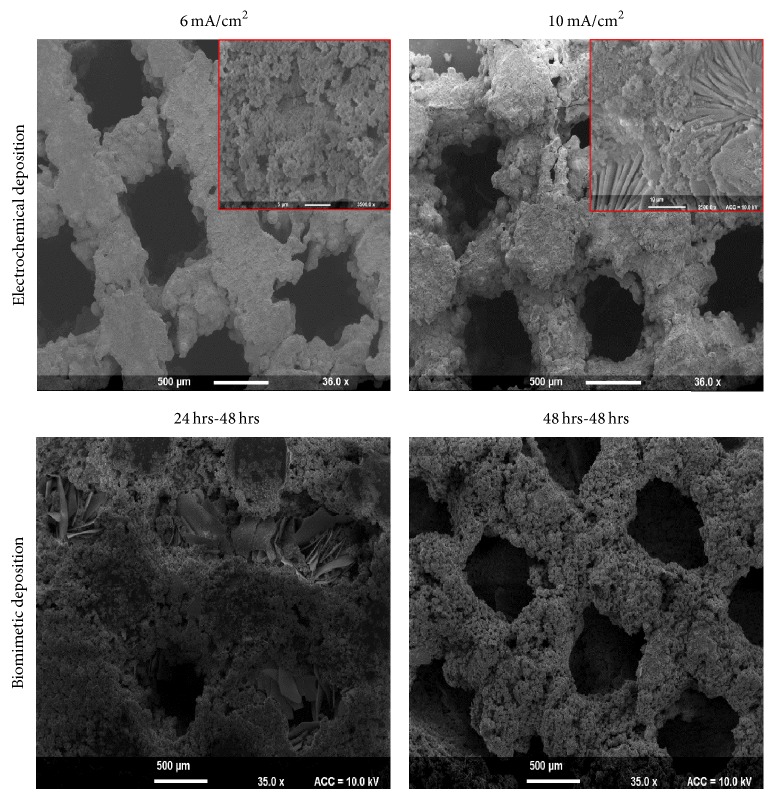
SEM micrographs of HAp coated Ti scaffolds using biomimetic and electrochemical methods.

Porous Ti matrices were coated with HAp using both a biomimetic and an electrochemical method in order to select the most suitable coating technique in terms of homogeneity. The morphology of the HAp coatings, examined by SEM, is shown in Figure 2 and the compositions of the coatings, determined by EDX analysis, are listed in Table 3.

From the EDX analysis, the molar ratio of calcium to phosphorous (Ca/P) of HAp was 1.79–1.85 for both coating methods showing a slightly calcium rich HAp compared to stoichiometric HAp (Ca/P = 1.67). While the Ca/P ratio was similar in all groups, it became apparent from SEM images that the coating was more uniform in electrochemical deposition method, whereas the biomimetic procedure usually led to nonuniform coating and blocking of the matrix pores.

Based on the uniformity criteria of the coatings, electrochemical method with 10 mA/cm^2^ setting (sample EM10) was chosen for tests on cell adhesion proliferation and mineralisation.

### 3.2. Mechanical Properties and In Vitro Fixation

The mechanical properties of Ti scaffold were determined in compression mode and are summarized in Table 4. It was observed that the Ti scaffolds exhibited a compressive strength of 35 MPa and Young's modulus of 0.55 GPa, which is within the range of trabecular bone (e.g., 1–100 MPa for compressive strength) [35, 36], and is comparable to values (24 MPa) achieved in a 75% porosity PM processed Ti matrix [19]. In terms of bone, mid-range values for the modulus of trabecular bone are 90–400 MPa. The compressive modulus of our Ti scaffold reached 73 MPa, which is slightly lower than the lower range of native bone modulus. However, it must be noted that the values of native bone vary considerably across different locations and patients. An example is the compression modulus of human cancellous bone obtained by Martens et al. (1983), where superior-anterior femoral head showed a modulus of 900 ± 714 MPa, while the anterior-posterior showed a modulus of only 12 ± 6 MPa and medial-lateral a modulus of 63 ± 7 MPa [37].

To evaluate the mechanical stability of scaffolds when placed in a defect, the push-in, push-out, and interfacial strengths as well as the push-in depth were assessed, and these are reported in Table 4. Mechanical stability can be influenced by interlocking of the newly formed bone, the macroscopic design of the implant, and its stiffness and interface stress, as well as the interface friction and the bone-implant gap size [38]. Because there is no contribution from ingrown bone, the stability of scaffold in vitro solely relies on the macroscopic design of scaffold, friction coefficient, and gap size between bone and scaffold.

In fact, friction influences the mechanical stability of an implant and its relative migration in the bone and affect seating of the implant in the bone [39]. The friction coefficient values obtained in Sawbones® in dry state are usually lower than those in the lubricated human bone resembling the actual implant condition.

We observed that the achieved push-in and push-out strengths were in fact greatly affected by the degree to which the scaffolds were fitted in the defects. Even slightest mismatch between geometries could lead to a significant decrease in the interface strength.

The push-in strength was determined to be significantly higher than the push-out strength (*p* = 0.004). It is believed that the tapered design of the scaffold contributed the higher values of push-in strength compared to push-out strength.

In a study on bone-implant interface strength and osseointegration in rats, the ex vivo interface shear strength of a Ti alloy implant after 4 weeks was reported to be in the range of 0.57–1.50 MPa [40]. The ultimate interface shear strength of porous Ti scaffold in our study is 0.81 MPa, demonstrating that the scaffold mechanical fixation in vitro is comparable to that of a Ti alloy implant in vivo and would fulfil one of the key requirements, that is, mechanical stability to be retained in the affected area, for bone scaffolds.

### 3.3. Evaluation of Osteoinductivity In Vitro

**Figure 3:**
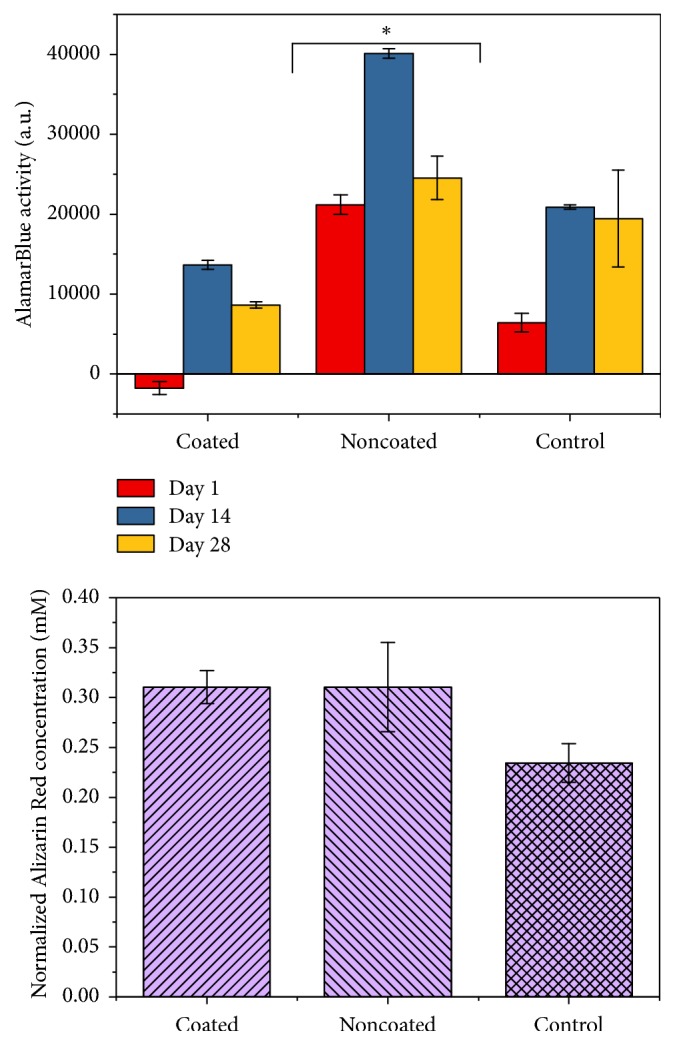
Biocompatibility and osteoinductivity of porous Ti scaffolds. AlamarBlue absorbance of BMMSCs seeded on HAp coated noncoated scaffolds over 28 days; Alizarin Red absorbance normalized to noncoated samples at day 28. *∗* shows significantly higher value compared to other samples (*p* < 0.05).

All scaffolds (HAp coated and noncoated) allowed for cell attachment and viability throughout 28-day culture period. Presence of metabolically active cells and their proliferation, as determined by AlamarBlue® analysis, indicated an increase in the number of cells attached on the scaffolds during the culture period (Figure 3). AlamarBlue activity was higher on day 1 on noncoated scaffolds compared to HAp coated scaffolds, showing higher cell attachment to noncoated samples. Metabolic activity peaked at day 14, again significantly higher on noncoated scaffolds, which may indicate higher cell division on these samples. The decrease in metabolic activity after day 14 may demonstrate stem cell differentiation towards an osteogenic lineage, which is confirmed by Alizarin Red quantification results on day 28.

**Figure 4:**
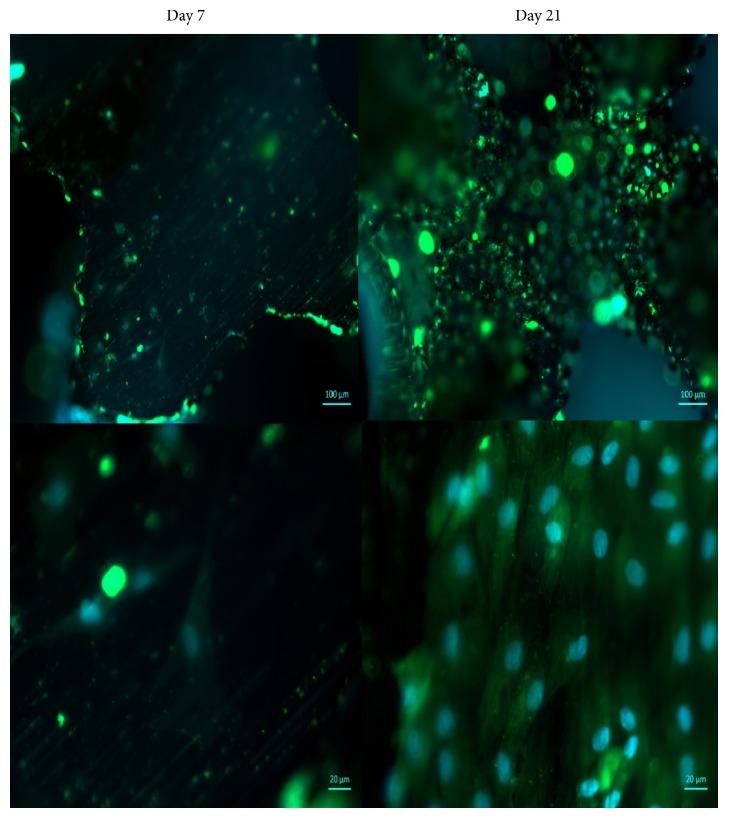
Cell-scaffold interaction; immunostaining of cells on porous noncoated Ti scaffold shows cell nuclei (DAPI, blue) and cytoskeleton (phalloidin, green) at days 7 and 21.

**Figure 5:**
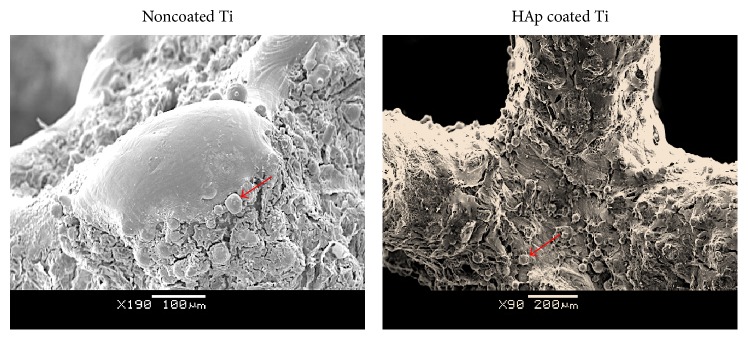
Cell morphology and osteogenesis on coated and noncoated Ti scaffolds; noncoated: flat cuboidal osteoblastic cells and mineral nodules (red arrow) on the scaffold peaks; coated: spindle-like cells and round mineral nodules (red arrow) attached to HAp coated scaffold surface.

Immunofluorescence staining for actin cytoskeleton showed a well-developed cytoskeleton suggesting cells could adopt a flattened and spread morphology on noncoated scaffolds, while DAPI staining indicated the presence of cells as evidenced by staining of their nuclei covering the entire scaffold surface (Figure 4). Scanning electron microscopy further confirmed the attachment and proliferation of the seeded cells on both coated and noncoated scaffolds. Spindle-like cells, with cell –cell contact were observed attached to the surface of the HAp coated and noncoated titanium scaffolds (Figure 5). Osteoblastic like cells and mineral nodules were observed on and within the core of the scaffolds by day 21 of the culture.

Alizarin Red staining was used to determine late-stage osteoblastic differentiation. Alizarin Red quantification over 21 days of culture period confirmed osteoblastic differentiation of the seeded cells on both coated and HAp noncoated scaffolds (Figure 3). Noncoated samples indicated higher Alizarin Red absorbance; however this may be associated with the poor release of the stain from the HA coating during the quantification process. By normalizing the concentrations to that of noncoated samples both HAp coated and noncoated scaffolds indicated similar levels of differentiation (Figure 3).

### 3.4. Bone Formation and Ingrowth

**Figure 6:**
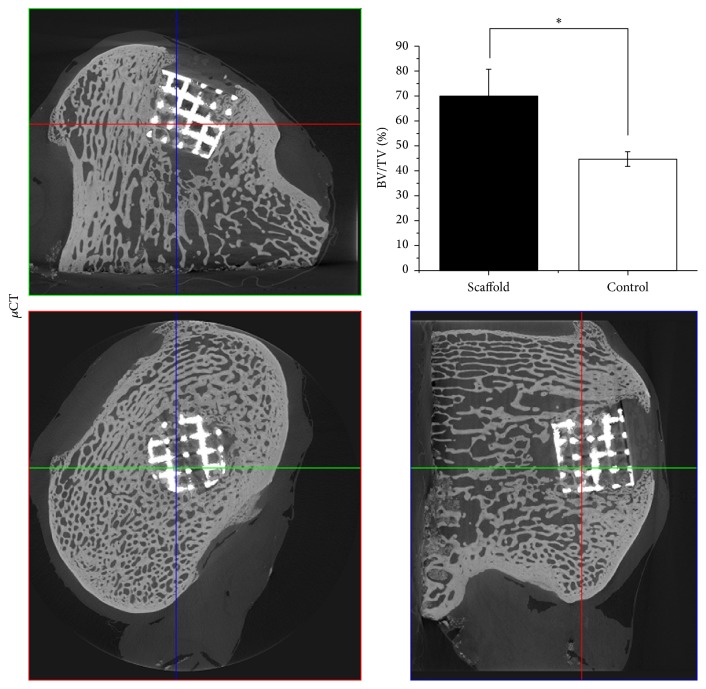
Bone ingrowth into Ti matrix. *μ*CT images show bone formation within the Ti scaffold; *∗* shows significantly higher bone volume formation in the scaffold compared to control (*p* = 0.01).

**Figure 7:**
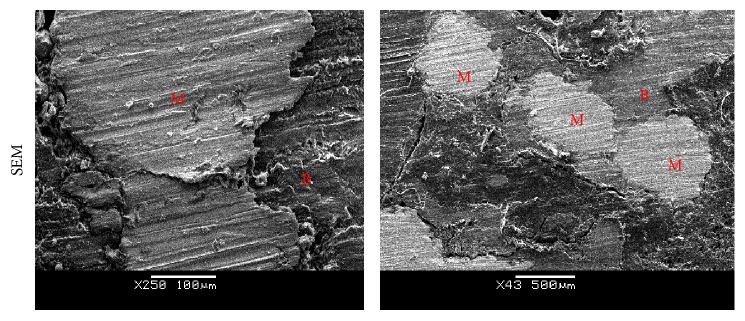
Bone-scaffold interface; SEM images show bone (B) in contact with the metal (M).

For the clinical determination of the bone ingrowth inside the scaffold recent advances in the *μ*CT imaging have shown sufficient resolution for the accurate identification of the bone ingrowth within the metallic porous structure [23]. A 3D volume of bone-scaffold was reconstructed from *μ*CT images and sagittal, coronal, and transverse sections are presented in Figure 6, showing bone trabeculae inside the scaffold porous structure. We observed areas of incomplete bone regeneration below the scaffold; the void is speculated to be generated during the surgery with the drill, which has been unable to heal. The amount of bone ingrowth in scaffold and control groups was quantified using the principles for bone histomorphometry and is reported in Figure 6. We observed that the BV/TV was significantly higher (*p* = 0.01) in the scaffold group compared to the control group, which consisted of a collagen-HAp matrix. The bone-implant contact was calculated to be 70%, and the interface between the Ti struts and the regenerated bone, demonstrating a very close contact between the two, is shown by SEM images in Figure 7, which further confirms integration of Ti scaffold and the newly formed bone.

We speculate that the significant increase in bone regeneration in the defects treated with porous titanium scaffolds compared to collagen-HAp scaffold may be related to the scaffold structure and its mechanical properties, as it possesses an interconnected porous structure and mechanical properties in range of trabecular bone. The mechanism of osteoinduction in porous biomaterials and its biological effects are still largely unknown. However, it has been argued that biomaterials must meet very specific requirements in terms of macrostructure (e.g., geometry and porosity), microstructure (e.g., microporosity and surface roughness), and chemical composition (calcification ability) in order to be osteoinductive [29, 41]. For example, it has been previously shown that porous Ti containing no calcium phosphate can become osteoinductive when it has a complex interconnecting porous structure and bioactive surfaces activated by simple chemical and thermal treatments [28, 29]. However, in this study, we have shown that surface modification and/or HAp coating might not be required for osteoinduction of porous titanium. The developed 3D printed Ti scaffold possess a rough surface (microstructure), an interconnected structure of pores with over 70% porosity, and mechanical properties in range of trabecular bone (macro- and microstructure), but they do not contain any source of calcium phosphate. In materials containing calcium phosphate, the Ca^2+^, PO^3−^_4_, and HPO^2−^_4_ are liberated from the surface into the surrounding which may increase the local supersaturation of the biologic fluid, causing precipitation of carbonated apatite that incorporates calcium, phosphate, and other ions [42]. The dissolution part of this process is missing in the materials that initially do not contain calcium phosphate; however, their physicochemical properties are such that they provide nucleation sites for the deposition of a biological apatite layer [41]. It is plausible that similar events occurred in the developed Ti scaffold providing nucleation sites for calcification and bone formation.

## 4. Conclusions

We have developed a highly porous (72%) Ti scaffold for bone tissue engineering using additive manufacturing (selective laser sintering) technique. The mechanical properties, including compressive strength and stiffness of the produced scaffold, were in range of human trabecular bone, and they showed a stable mechanical fixation in vitro, comparable to fixations observed after 4 weeks of bone ingrowth. We showed that both HAp coated and noncoated scaffolds promote osteogenesis in vitro and HAp coating did not produce a significant increase in late osteogenesis. Bone formation and ingrowth in sheep stifle joints confirmed that the Ti scaffolds—as produced and without any coating—exhibited an intrinsic capacity for bone formation and osteoinduction. Therefore, these scaffolds have the potential to be used for tissue engineering of large bone defects and nonunions.

## Figures and Tables

**Table 1:** Composition of SBF coating solutions.

	NaCl	NaHCO_3_	K_2_HPO_4_·3H_2_O	MgCL_2_·6H_2_O	CaCl_2_·2H_2_O
Solution A	680 mM	21 mM	5 mM	7.6 mM	9.9 mM
Solution B	680 mM	10 mM	5 mM	1.5 mM	9.9 mM

**Table 2:** Scaffold formulations in terms of coating technique and conditions.

Coating method	Condition		Acronym
Biomimetic method	Solution A (hrs)	Solution B (hrs)	
	24	48	BM24
	48	48	BM48

Electrochemical method	Current density (mA/cm^2^)		
	6	10	EM6 and EM10

**Table 3:** Composition (Ca/P ratio) of different coatings obtained by EDX; *∗* is used for further cell analyses.

Sample	Ca/P ratio
EM6	1.84 ± 0.54
EM10^*∗*^	1.85 ± 0.25
BM24	1.79 ± 0.21
BM48	1.85 ± 0.08

**Table 4:** Mechanical properties of porous Ti scaffold and its mechanical fixation in Sawbones. Data are expressed as mean ± standard deviation; *∗* shows significant difference at *p* = 0.004.

Mechanical characteristics	
Compressive strength	35 MPa
Compressive modulus	73 MPa
Young's modulus	0.55 MPa

Mechanical stability in vitro	

Push-in depth	5.8 mm
Push-in strength	5.67 MPa (±1.54)^*∗*^
Push-out strength	1.44 MPa (±1.14)^*∗*^
Interfacial shear strength	0.81 MPa (±0.36)
